# Mutational effects of γ-rays and carbon ion beams on *Arabidopsis* seedlings

**DOI:** 10.1093/jrr/rrt074

**Published:** 2013-05-31

**Authors:** Ryouhei Yoshihara, Shigeki Nozawa, Yoshihiro Hase, Issay Narumi, Jun Hidema, Ayako N. Sakamoto

**Affiliations:** 1 Research Center for Environmental Genomics, Kobe University, 1-1 Rokkodai, Nada, Kobe 657-8501, Japan; 2 Ion Beam Mutagenesis Research Group, Medical and Biotechnological Application Division, Quantum Beam Science Directorate, Japan Atomic Energy Agency, 1233 Watanuki, Takasaki, 370-1292, Japan; 3 Department of Life Sciences, Faculty of Life Sciences, Toyo University, 1-1-1 Izumino, Itakura, 374-0193, Japan; 4 Graduate School of Life Sciences, Tohoku University, 2-1-1 Katahira, Aoba-ku, Sendai 980-8577, Japan

**Keywords:** mutation spectrum, γ-rays; carbon ion beams, *Arabidopsis*, *rpsL* gene

## Abstract

To assess the mutational effects of radiation on vigorously proliferating plant tissue, the mutation spectrum was analyzed with *Arabidopsis* seedlings using the plasmid-rescue method. Transgenic plants containing the *Escherichia coli rpsL* gene were irradiated with γ-rays and carbon ion beams (320-MeV ^12^C^6+^), and mutations in the *rpsL* gene were analyzed. Mutant frequency increased significantly following irradiation by γ-rays, but not by 320-MeV ^12^C^6+^. Mutation spectra showed that both radiations increased the frequency of frameshifts and other mutations, including deletions and insertions, but only γ-rays increased the frequency of total base substitutions. These results suggest that the type of DNA lesions which cause base substitutions were less often induced by 320-MeV ^12^C^6+^ than by γ-rays in *Arabidopsis* seedlings. Furthermore, γ-rays never increased the frequencies of G:C to T:A or A:T to C:G transversions, which are caused by oxidized guanine; 320-MeV ^12^C^6+^, however, produced a slight increase in both transversions. Instead, γ-rays produced a significant increase in the frequency of G:C to A:T transitions. These results suggest that 8-oxoguanine has little effect on mutagenesis in *Arabidopsis* cells.

## INTRODUCTION

The biological effect of ionizing radiation has been studied in bacteria and animals over many decades. Ion beams and γ-rays are utilized as models of high-linear energy transfer (LET) and low-LET radiation, respectively, to study the biological effects from various radiations. It is known that high-LET radiations tend to confer greater biological effects (such as cell-killing and mutagenesis) than low-LET radiations [1–3]. It is thought that the greater cell-killing effects of high LET radiation are due to the formation of double-strand breaks (DSBs) and clustered DNA damage, which are deleterious for organisms [4, 5]. Mutations induced by high- and low-LET radiations are also of different types. It has been reported for mammalian cells that ion beams induce large-sized deletions and insertions, and chromosomal rearrangements, whereas γ-rays induce shorter deletions and insertions, and more frequent base substitutions [3].

Several studies have reported that high-LET radiations confer greater and more distinctive biological effects on plant cells than low-LET radiations. For instance, high-LET radiations produce a more lethal effect on *Arabidopsis* and tobacco cells [6–9]. As for the types of mutation, irradiation of *Arabidopsis* dry seeds by carbon ion beams induces point-like mutations (base substitutions and several-base deletions/insertions) and chromosomal rearrangements with similar frequencies, whereas electron beams, one of the low-LET forms of radiation, predominantly induce point-like mutations [10, 11]. Furthermore, genomic *in situ* hybridization in wheat has shown that ion beams induce chromosomal rearrangements more frequently than X-rays [12]. Recently, Hase *et al.* (2012) showed that high-LET ion beams induce DSBs that are seldom repaired by the non-homologous end-joining (NHEJ) pathway in *Arabidopsis* dry seed [13]. These results suggest that the significant biological effects of high-LET radiation on plants seem to be due to differences in DNA damage, as reported in other organisms.

In general, ionizing radiation predominantly induces the formation of radicals by water radiolysis, and these then attack DNA and produce oxidative damage. The formation of 8-oxogunanine is one of the major forms of radiation-induced oxidative damage in animal and bacterial cells, and it is believed to induce major base substitutions involving G:C to T:A and A:T to C:G transversions [14]. The oxidative DNA damage causes mispairings during DNA replication [15].

In a prior study, we irradiated *Arabidopsis* dry seeds with carbon ion beams and γ-rays to analyze radiation-induced mutation in *Arabidopsis* somatic cells. Our results showed no significant increase of G:C to T:A or A:T to C:G transversions with either type of radiation, suggesting the low effect of 8-oxoguanine in *Arabidopsis* dry seeds [16]. This may be because the low water content and/or low cell proliferation activity in dry seed help to avoid the effect of 8-oxoguanine and other oxidative damage. In this study, we analyzed the mutations induced by carbon ion beams (320-MeV ^12^C^6+^) and γ-rays in *Arabidopsis* seedlings. This is the first report detecting radiation-induced mutations in vigorous plant cells with high water content and high cell proliferation activity. Our results provide an important insight for evaluation of the mutational effects of ionizing radiation on plant systems.

## MATERIALS AND METHODS

### Plant and bacterial strains

For mutation spectrum analysis, we used transgenic *Arabidopsis* containing the *E. coli rpsL* gene integrated into the chromosomal DNA [17]. Mutations occurring in the *rpsL* gene were detected by means of the plasmid-rescue technique. *E. coli* strain DH10B [F^−^ *mcrA Δ*(*mrr-hsdRMS*-*mcrBC*) φ80d *lacZΔM15 ΔlacX74 deoR recA1 endA1 araD139 Δ*(*ara leu*)7697 *galU galK rpsL nupG* λ^−^] carrying the *supF*-containing plasmid pFSE101 (DH10B/pFSE101) was used for the screening of *rpsL* mutant clones [18].

### Ionizing radiation

Transgenic *Arabidopsis* seedlings were irradiated with carbon ion (^12^C^6+^) beams generated using an azimuthally varying field (AVF) cyclotron and ^60^Co γ-rays (Japan Atomic Energy Agency, Takasaki, Gunma, Japan). The energy of a carbon ion beam was 320-MeV (26.7 MeV/u) and the average LET was 86.2 keV/μm, which was calculated using the ELOSSM code, a program used to calculate the energy loss of heavy ions under practical irradiation conditions [19].

### Plant cultivation and irradiation

The *rpsL*-transgenic plants were sown on 1/2 B5 medium (1/2 × Gamborg's B5 salts, 1/1000 × hyponex, 1% sucrose, 0.7% agar) aseptically. After a three-day 4°C treatment, plants were grown at 23°C under a 16-h light and 8-h dark cycle for 7 days. For mutation spectrum analysis, 7-day-old plants were irradiated with carbon ions or γ-rays. Irradiated plants were grown under the same conditions for another 7 days and used for mutation analysis.

### Sensitivity to ionizing radiation

Sensitivity to ionizing radiation was evaluated by measuring the area of the fifth leaf of irradiated 14-day-old plants. The leaves were mounted on a paper and the leaf area was measured using Adobe Photoshop Elements 4.0 software (Adobe systems Inc., San Jose, CA, USA). Radiation sensitivity was determined as the relative leaf size of irradiated leaves to that of non-irradiated control leaves. Sensitivity curves fitted using a least-square method were defined by the following equation:

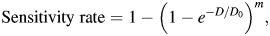

where *D*, *D*_*0*_ and *m* indicate the dose, the dose conferring a 37% sensitivity rate (mean lethal dose), and the extrapolated number (the number of targets), respectively.

### Mutation analysis

We used ∼270 plants per experimental plot. The experiment was repeated at least five times. Chromosomal DNA was isolated by the CTAB method [20]. Approximately 80 µg of DNA was digested with 20 units of *Bam*HI (Takara Bio, Otsu, Shiga, Japan) in a 400-μl reaction mixture. Digested DNA was purified by phenol/chloroform extraction and ethanol precipitation. The DNA was self-ligated in an 800-μl reaction mixture containing 11 Weiss units of T4 DNA ligase (Takara Bio). Reconstructed plasmid purified by phenol/chloroform extraction and ethanol precipitation was electroporated into *E. coli* DH10B/pFSE101. To select clones carrying a mutated *rpsL* gene, this *E. coli* was screened on agar medium containing 100 µg/ml of kanamycin (Km) and 60 µg/ml of streptomycin (Sm). The number of total clones was determined by plating a portion of electroporated cells on the medium containing 100 µg/ml of Km. Mutant frequency was calculated as the ratio of mutant clone to total clone. Sequence analysis was performed using a GenomeLab GeXP Genetic Analysis System (Beckman Coulter Inc., Brea, CA, USA).

We analyzed a total of 3.1–20 × 10^4^ clones in each rescue experiment and repeated experiments at least five times. Plural mutations in one clone, i.e. multiple base changes, a base change combined with a frameshift, or a deletion accompanied with an insertion, were scored as a ‘complex mutation’. All mutations from independent clones were counted as independent mutations.

## RESULTS

### Sensitivity of *Arabidopsis* seedlings to γ-rays and 320-MeV ^12^C^6+^

**Fig. 1.**
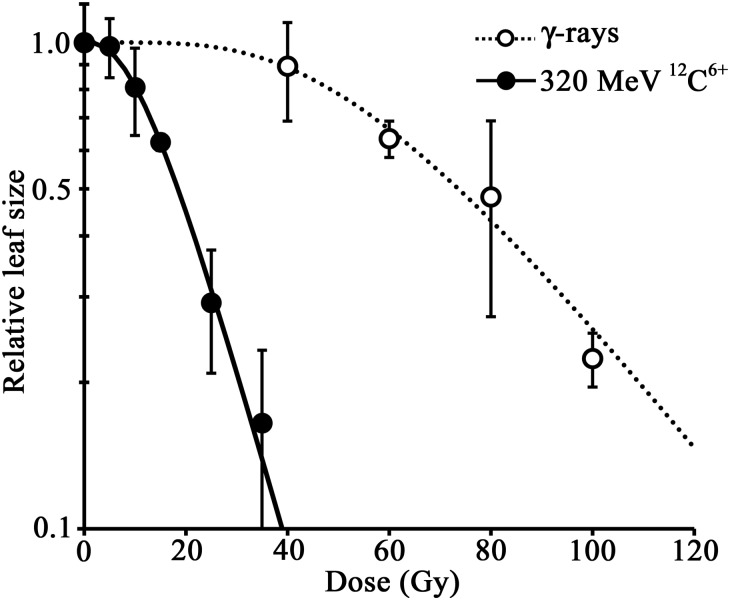
Radiation sensitivity of *rpsL*-transgenic *Arabidopsis*. The effect of γ-rays (open circles) and 320-MeV ^12^C^6+^ ion beams (filled circles) on the growth of *Arabidopsis* leaves. Radiation sensitivity is shown as the relative leaf size of irradiated plants to that of the unirradiated control plants. Error bars represent the standard deviation of the mean.

To compare the effects of γ-rays and 320-MeV ^12^C^6+^ ion beams on plant tissues, the growth inhibition on young plant tissue was quantified. Seven-day-old seedlings, on which 1–2 true leaves have emerged, were irradiated with γ-rays and 320-MeV ^12^C^6+^ ion beams. The plants were grown for another 7 days, by which time the plants had developed 4–6 true leaves. We cut the fifth leaf from each plant and measured the area of the leaf using graphics software. Results showed that 320-MeV ^12^C^6+^ more severely inhibited the growth of plant leaves than the same dose of γ-rays (Fig. 1). This is due to the deleterious effect of 320-MeV ^12^C^6+^ on cell proliferation. To standardize the effect of both radiations, we adopted the Multi-Target Single-Hit model (Fig. 1) that has been shown to work well in plants [7, 9, 16]. Based on these sensitivity curves, 40–100 Gy of γ-rays and 5–35 Gy of 320-MeV ^12^C^6+^ reduced the leaf size dose-dependently. We expected that these ranges of radiations might induce mutations effectively in the plant. Based on this prediction, we chose the dose of 64 Gy and 20 Gy for γ-rays and 320-MeV ^12^C^6+^, respectively, which reduced the leaf size to ∼50%, and then conducted mutation spectrum analysis.

### Mutagenicity of γ-rays and 320-MeV ^12^C^6+^ ion beams

To investigate the mutational effect of γ-rays and 320-MeV ^12^C^6+^ ion beams on *Arabidopsis* seedlings, the mutation frequencies were determined using the plasmid-rescue method (Table 1). The mutant frequency of unirradiated seedlings was 3.4 × 10^−5^, as found in our previous report [16]. By irradiating with 64 Gy of γ-rays, the mutant frequency increased significantly (4.1-fold, *P* < 0.01). In contrast, 20 Gy of carbon ions increased the mutant frequency by 2.9-fold, but this increase was not statistically significant. Therefore, these results suggest that a 320-MeV ^12^C^6+^ ion beam is less effective than γ-rays in inducing detectable mutations in this assay system.

**Table 1.** Mutant frequencies induced by γ-rays and 320-MeV ^12^C^6+^ ion beams

	Mutant clone^a^	Total clone^a^ (×10^5^)	Mutant frequency^b^ (×10^−5^)
Background	33	9.2	3.4 ± 0.8
γ-rays	44	3.6	14 ± 7*
320-MeV ^12^C^6+^ ion beams	27	3.3	10 ± 12

^a^Sum of 5–10 independent experiments. ^b^Average of 5–10 independent experiments with SD. *Statistically significant compared to unirradiated (‘background’) plants (*P* < 0.01).

### Mutation induced by γ-rays and a 320-MeV ^12^C^6+^ ion beam in *Arabidopsis* seedlings

**Fig. 2.**
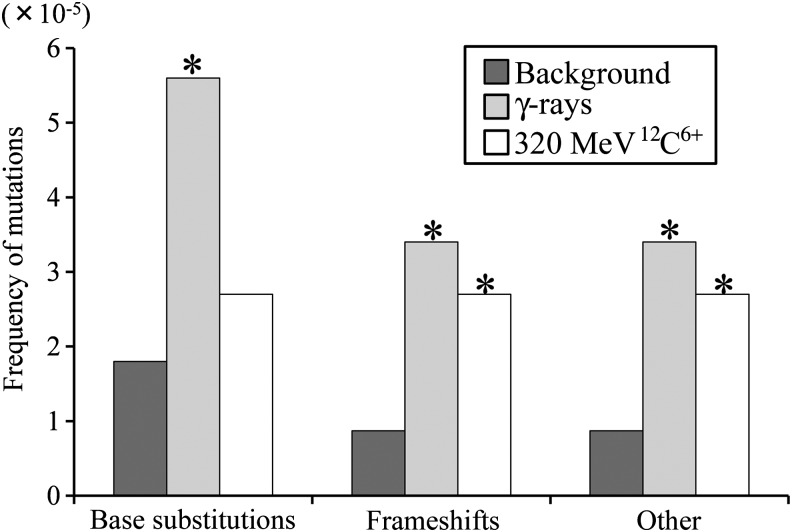
Frequency of mutations classified into three categories. The frequency of mutations was calculated from the ratio of mutant clone numbers to predicted total analyzed clone numbers. Frameshifts include one base insertion, one base deletion, and deletion of two contiguous bases. Others are a deletion or insertion of ≥3 bases, plural base substitutions, base substitution accompanied by a frameshift, and deletion accompanied by an insertion. *Statistically significant compared with unirradiated (‘background’) plants according to the Poisson test (*P* < 0.01).

We analyzed mutation spectra induced by γ-rays and a 320-MeV ^12^C^6+^ ion beam, as well as unirradiated (‘background’) seedlings. All mutations were classified into three groups: base substitutions, frameshifts, and ‘other’ (Fig. 2). In comparison with unirradiated plants, both radiations increased the frequency of frameshifts and ‘other’ mutations, but only γ-rays resulted in a significant increase in the frequency of total base substitutions.

We analyzed the type of each mutation (Table 2). Regarding the types of base substitutions, γ-rays resulted in a significant increase in the frequency of G:C to A:T transitions (*P* < 0.01). Furthermore, γ-rays increased the frequencies of –1 and –2 frameshifts, deletions/insertions, and complex mutations (*P* < 0.01). In contrast, 320-MeV ^12^C^6+^ increased G:C to T:A and A:T to C:G transversions, –1 frameshifts and complex mutations (*P* < 0.01). There was no increase in both transversions associated with γ-rays, which was consistent with our previous mutation analysis in *Arabidopsis* dry seeds [16].

**Table 2.** Mutation spectra of *Arabidopsis* seedlings following ionizing radiation

	Background		γ-rays		320-MeV ^12^C^6+^	
	NM	MF (×10^−5^)	NM	MF (×10^−5^)	NM	MF (×10^−5^)
Transition						
G → A	9	(1.0)	11	(3.1)*	2	(0.6)
A → G	1	(0.1)	0	(<0.3)	0	(<0.3)
Transversion						
G → T	1	(0.1)	2	(0.6)	3	(0.9)*
G → C	3	(0.3)	2	(0.6)	2	(0.6)
A → T	3	(0.3)	4	(1.1)**	0	(<0.3)
A → C	0	(<0.1)	1	(0.3)	2	(0.6)*
Frameshifts						
+1	0	(<0.1)	1	(0.3)	1	(0.3)
–1	8	(0.9)	9	(2.5)*	8	(2.4)*
–2	0	(<0.1)	2	(0.6)*	0	(<0.3)
Deletion	8	(0.9)	9	(2.5)*	6	(1.8)**
Complex	0	(< 0.1)	3	(0.8)*	3	(0.9)*
Total	33	(3.6)	44	(12.4)	27	(8.2)

Mutant frequency is derived from the ratio of mutant clone numbers to predicted total analyzed clone numbers. NM = number of mutants, MF = mutant frequency. The statistical significance of differences between the irradiated group and the unirradiated (‘background’) group was examined using the Poisson test (*P < 0.01, **P < 0.05).

## DISCUSSION

In this study, we irradiated *Arabidopsis* seedlings with 64 Gy of γ-rays and 20 Gy of 320-MeV ^12^C^6+^ ion beams, which reduced leaf size to ∼50%, then analyzed the mutation spectrum. In the previous study, we irradiated Arabidopsis dry seeds with 740 Gy of γ-rays and 140 Gy of 208-MeV ^12^C^5+^, each of which correspond to 0.8 Dq of survivals and provide similar mutation frequencies (Yoshihara *et al*, 2010). Here, we used the reduction of leaf size as an index and expected the doses causing a similar degree of reduction to also cause a similar induction of mutation frequency. However, results revealed that the mutant frequency increased following irradiation by γ-rays (64 Gy), but did not increase significantly following irradiation by 320-MeV ^12^C^6+^ (20 Gy), although both conditions reduced leaf size to ∼50%. Since the growth of leaves depends on multiple factors, such as cell death, cell-cycle arrest, or cell-expansion, it did not simply correlate with the DNA damage and/or mutations. Namely, the reason that the 320-MeV ^12^C^6+^ ion beam did not elevate mutant frequency could be because the dose was too low to induce mutations. It would be necessary to analyze the dose response of mutations in order to elucidate all the characteristics of these radiations. Nevertheless, though limited, the data obtained in this work shed light on the understanding of mutations by γ-rays and a 320-MeV ^12^C^6+^ ion beam, as discussed below.

The mutation spectrum analysis showed an increase in the total number of base substitutions after γ-ray irradiation, but not after radiation from the 320-MeV ^12^C^6^^+^ ion beam (Fig. 2). Oxidative damage to DNA, which is mainly caused by radicals from water radiolysis as an indirect effect of ionizing radiation, is often involved in the induction of base substitutions [21]. Therefore, it is possible that more radicals were induced in plant cells by γ-rays than by the 320-MeV ^12^C^6+^ ion beam in these assay conditions. This difference might have caused the difference in mutant frequencies.

In contrast, both types of radiations increased the frequency of deletions/insertions and complex mutations compared with that observed in unirradiated plants (Fig. 2). It is known that these mutations occur during the process of DSB repair by non-homologous end-joining (NHEJ) [22]. It is reasonable to claim that both radiations induced DSBs in *Arabidopsis* plants. In our previous work, *Arabidopsis* plants with a disrupted DNA Ligase IV gene, a key component of the NHEJ pathway, showed a markedly higher sensitivity than the wild-type to ion beams [13]. These data also support the idea that DSBs formed by the radiation exposure were mostly repaired by the NHEJ pathway in *Arabidopsis* seedlings. The simplest interpretation is that the γ-rays and 320-MeV ^12^C^6+^ carbon beam induced a comparable number of DSBs, which led to deletions/insertions or complex mutations with similar frequencies (3.4 and 2.7 × 10^−5^, respectively) (Fig. 2).

In this study, plural base substitutions, base substitution accompanied by a frameshift, and a deletion combined with an insertion, were categorized as ‘complex mutations’. These mutations are known to be induced by repair or replication of damaged DNA. For example, in the NHEJ repair process, 1–2 bp of microhomology near the DNA end is often utilized to rejoin the DSB. After annealing of the homologous base(s), the flanking mismatched base(s) is sometimes deleted or inserted by fill-in activity [23, 24]. Some DNA polymerase can fill the strand gap even when the template has a gap, a damaged base, or a mismatched base [25–27]. Such error-prone repair may lead to a deletion combined with an insertion, or a base substitution accompanied by a frameshift, which is likely to occur when the DSB end is not complementary. It is known that a template involving a damaged base causes primer–template misalignment, which may induce plural base substitutions or a base substitution accompanied by a frameshift [28, 29]. Such error-prone replications are likely to occur when DNA damage is not completely removed before replication. The mutation spectra showed that both γ-rays and 320-MeV ^12^C^6+^ ion beams significantly induced complex mutations (Fig. 2). We therefore speculate that some DSBs, or other base damage induced by both radiations, are difficult to repair, and that the plant cells are subject to error-prone repair or replication.

Both γ-rays and 320-MeV ^12^C^6+^ ion beams increased the frequency of –1 frameshift mutations (Table 2). It is thought that +1 and –1 frameshifts are mainly caused by primer–template misalignment at or near the damaged base during DNA replication [30–32]. In addition, it has been shown that some +1 and –1 frameshifts may be induced in the process of DSB repair by NHEJ [33, 34]. In yeast, it was estimated that ∼50% of +1 and –1 frameshifts are induced by the NHEJ pathway [34]. Considering the few base substitutions induced by the 320-MeV ^12^C^6+^ ion beams (Fig. 2), it is possible that the formation of base lesions was not elevated by 320-MeV ^12^C^6+^ ion beams compared with that of unirradiated plants. In contrast, the frameshifts were significantly higher in 320-MeV ^12^C^6+^ ion beam-irradiated plants than in unirradiated plants (Fig. 2). Therefore, it is likely that the increase in –1 frameshifts in irradiated seedlings was due to failed DSB repair by the NHEJ pathway, at least in part.

The G:C to T:A and A:T to C:G transversions are mainly induced by guanine oxidation and subsequent 8-oxoguanine and adenine mispairing during DNA replication [15]. Since *Arabidopsis* seedlings have a high water content and cell proliferation activity, we predicted that the mutations in seedlings might reflect the increased 8-oxoguanine and other oxidative damage caused by the irradiation. Contrary to our prediction, however, γ-rays did not increase the G:C to T:A and A:T to C:G transversions in *Arabidopsis* seedlings (Table 2). In contrast, 320-MeV ^12^C^6+^ ion beams increased both transversions by 8.4 and >5.6 times, which is statistically significant compared with control seedlings. However, the total numbers of G:C to T:A and A:T to C:G transversions were only 3 and 2, respectively, which were fewer than those of other mutations such as –1 frameshifts and deletions/insertions in 320-MeV ^12^C^6+^ ion beam-irradiated plants. It is premature to conclude that 320-MeV ^12^C^6+^ ion beams induced more 8-oxoguanine than γ-rays in plant cells. Instead, as mentioned below, it is possible that various forms of oxidative damage other than 8-oxoguanine are major sources of mutations in γ-ray-irradiated plants. It is reasonable to expect that 8-oxoguanine has a limited effect on mutagenesis in *Arabidopsis* cells.

The important repair enzymes for 8-oxoguanine in *E. coli* and humans are *mutT/MTH1*, *mutY/MYH* and *mutM/OGG1* [35, 36]. The *Arabidopsis* genome also has the homologs of these genes [37]. However, mutant plants deficient in the *mutT/MTH1-* or *mutM/OGG1*-homolog showed indistinguishable sensitivity to oxidative stress compared with wild-type plants [38, 39]. Our preliminary experiment showed that the γ-ray sensitivity of *Arabidopsis mth1* and *myh1* mutant plants was the same as that of wild-type plants (data not shown). On the basis of these findings, it is possible that (i) ionizing radiation causes only a modest increase in the amount of 8-oxoguanine in *Arabidopsis* cells, or (ii) *Arabidopsis* has another system(s) to suppress the mutagenic effect of 8-oxoguanine.

The frequency of G:C to A:T transitions increased significantly following γ-ray exposure (Table 2). The G:C to A:T transition is known to be induced by various types of base lesions. Cytosine glycol, a form of oxidized cytosine, is a possible candidate lesion that induced G:G to A:T transitions in *Arabidopsis* seedlings. Cytosine glycol often changes to 5-hydroxy-uracil by dehydration and deamination, which is mispaired with adenine and induces the G:C to A:T transition [14].

Uracil DNA glycosylase (Ung) and single-strand selective monofunctional uracil DNA glycosylase (Smug1) excise oxidized cytosine derivatives from DNA [14]. An *et al.* (2005) showed that these enzymes have redundant roles in resisting the effects of γ-rays and preventing G:C to A:T transitions in mammalian cells [40]. In addition, mammalian cells have thymine DNA glycosylase (TDG), endonuclease III (Nth) and endonuclease VIII (Nei), and *E. coli* cells have Ung, Nth, Nei and mismatch-specific uracil DNA-glycosylase (MUG) as glycosylases for oxidative cytosine derivatives [14]. Only Ung and Nth are found in *Arabidopsis* [41, 42]. Therefore, it is possible that *Arabidopsis* has a weak repair ability for oxidative cytosine derivatives and cannot prevent G:C to A:T transitions after exposure to γ-rays.

## CONCLUSION

In conclusion, our mutation spectrum analysis suggested that plants might have a unique mechanism for maintaining genomic stability against radiation-induced oxidative damage. Further studies, including dose–response analysis of mutagenesis for both radiations, are needed to elucidate the details of the DNA repair pathways and radiation mutagenesis in higher plants.

## FUNDING

This work was supported by the Atomic Energy Basic Infrastructure Research Initiative (210105) from the Japan Science and Technology Agency (JST), as well as Grants-in-Aid for Scientific Research (A) (24241028) and Scientific Research (C) (22570055) from the Japan Society for the Promotion of Science (JSPS).
